# Beneficial self‐management support and user involvement in Healthy Life Centres—A qualitative interview study in persons afflicted by overweight or obesity

**DOI:** 10.1111/hex.13129

**Published:** 2020-08-30

**Authors:** Elin Salemonsen, Georg Førland, Britt Sætre Hansen, Anne Lise Holm

**Affiliations:** ^1^ Department of Health and Caring Science Faculty of Health and Social Sciences Western Norway University of Applied Sciences Haugesund Norway; ^2^ Faculty of Health Sciences University of Stavanger Stavanger Norway

**Keywords:** dignity, empowerment, lifestyle change, long‐term individualized support, obesity, overweight, self‐efficacy, self‐management support, user involvement

## Abstract

**Background:**

Relapse is high in lifestyle interventions involving behavioural change and weight loss maintenance. The purpose of lifestyle self‐management interventions offered at Healthy Life Centres (HLCs) is to empower the participants, leading to self‐management and improved health. Exploring beneficial self‐management support and user involvement in HLCs is critical for quality, improving effectiveness and guiding approaches to lifestyle change support in overweight and obesity.

**Objective:**

The aim of this study was to explore how persons afflicted by overweight or obesity attending lifestyle interventions in Norwegian HLCs experience beneficial self‐management support and user involvement.

**Method:**

Semi‐structured in‐depth interviews were conducted with 13 service users (5 men and 8 women). Data were analysed using qualitative content analysis.

**Results:**

One main theme was identified: regaining self‐esteem and dignity through active involvement and long‐term self‐worth support in partnership with others. This main theme comprised four themes: (a) self‐efficacy through active involvement and better perceived health, (b) valued through health‐care professionals (HPs) acknowledgement, equality and individualized support, (c) increased motivation and self‐belief through fellowship and peer support; and (d) maintenance of lifestyle change through accessibility and long‐term support.

**Conclusion:**

Service users’ active involvement, acknowledgement and long‐term self‐worth support from HPs and peers seem to support self‐management and user involvement and may be some of the successful ingredients to lifestyle change. However, prolonged follow‐up support is needed. A collectivistic and long‐term perspective can integrate the importance of significant others and shared responsibility.

**Abbreviations**
HLC: Healthy Life Centres
HPs: health‐care professionals
NCDs: non‐communicable diseases

## 1 BACKGROUND

Overweight and obesity are considered some of the primary drivers of chronic non‐communicable diseases (NCDs), such as cardiovascular diseases, cancer, diabetes type 2 and chronic respiratory conditions.^1^ Worldwide, more than 1.9 billion adults are afflicted by overweight, of whom 650 million present with obesity.^2^ Due to this high prevalence, overweight and obesity have become a significant national and international health concern and place an extensive burden on health‐care services worldwide.^1^, ^2^, ^3^

The vast burden on health‐care services has led to the increased development of educational self‐management interventions.^4^, ^5^, ^6^ These behavioural interventions aim to help patients and service users better manage their own conditions (self‐care) and health‐care needs.^3^, ^4^, ^6^ Self‐management may be one means of bridging the gap between patients’ needs and the capacity of health‐care services to meet those needs.^5^ An individual's ability to detect and manage symptoms, treatment, physical and psychosocial consequences, and the lifestyle changes (such as exercise and diet) inherent in living with a chronic condition is the core of self‐management.^5^ In this study, self‐management support is understood as interventions or educational approaches supporting self‐management.^4^, ^6^ The desired outcome of self‐management support is behavioural change.^7^

There has been a growing interest in national and international health policies to more actively involve and empower patients in their health care.^8^, ^9^, ^10^ Individual empowerment is a process through which people gain greater control over decisions and actions affecting their health.^11^ The literature highlights patient and user involvement as a means of ensuring the quality of care and health services.^12^, ^13^ In this study, user involvement is understood as a clinical partnership between the service user and health‐care professionals (HPs).^13^ According to Greenhalgh, structured self‐management programmes focusing on building patients’ self‐efficacy can be seen as synonymous with patient or user involvement in managing chronic diseases.^13^

The literature on lifestyle change and self‐management interventions in chronic conditions or NCDs is extensive. Systematic reviews and meta‐analysis demonstrate that both individual and group‐based interventions designed to target dietary and physical activity behaviours are recommended strategies for lifestyle change^14^, ^15^ and weight loss maintenance.^16^, ^17^

The literature in the field of obesity is scarce regarding patient education and self‐management interventions, while self‐management terminology is rarely used in the context of overweight and obesity. One review and one meta‐analysis found in the literature on patient education about obesity suggest that patient health outcomes, including self‐management skills and quality of life, could be improved.^18^, ^19^ However, these analyses are based on the literature related to specific NCD diagnoses such as diabetes and not on generic programmes targeting educational interventions for overweight and obesity per se. The main challenge of overweight and obesity treatment is not merely weight loss, but long‐term weight loss maintenance.^17^, ^20^, ^21^, ^22^ Most efforts to change behaviours have limited success.^23^, ^24^ Findings provide initial evidence that overlooking psychosocial factors, such as weight stigma, may hinder weight loss maintenance and hamper help‐seeking.^25^, ^26^, ^27^

The Norwegian Directorate of Health recommends the establishment of Healthy Life Centres (HLCs) in primary health care to meet the challenge of overweigh, obesity and NCDs. HLCs offer lifestyle self‐management interventions for persons in need of support for lifestyle change who already have or are at risk of NCDs. The purpose of the intervention is to empower the participants, leading to self‐management and improved health.^28^ HLCs are a novelty in primary health care and health service research; thus, the knowledge of their efficacy is sparse. One study shows that less physically active persons became more physically active after attending a HLC.^29^ Both physical fitness and health‐related quality of life improved significantly in the short and the long term,^30^ and at the 24‐month follow‐up, no one had developed type 2 diabetes.^31^ One qualitative study from a HLC found that having a trustful relationship with the HPs being respected and experiencing continuity in the care were essential for service user involvement.^32^ Another qualitative study highlighted social support and pointed out the need for more research on how HPs in HLCs can help with and promote lasting lifestyle changes and whether HLCs can help participants who want and need such changes.^33^ However, no studies exploring what the participants really find beneficial when they seek help to change their lifestyle at a HLC have been found, and little is known about the significance of user involvement for lifestyle change in overweight and obesity care. Knowledge about the service users’ experiences of self‐management support as well as how aspects of user involvement affect participation in interventions could be helpful in understanding the overall process of lifestyle change, and how HLCs may provide beneficial support and a qualitative good health‐care service. Such knowledge is highly relevant for the future development of lifestyle interventions in HLCs. Thus, the aim of this study was to explore how persons afflicted by overweight or obesity attending lifestyle interventions in Norwegian HLCs experience beneficial self‐management support and user involvement.

## 2 METHODS

### 2.1 Design

A qualitative, interpretative interview study, grounded in hermeneutic tradition, was designed to explore beneficial self‐management support and user involvement for the service users in HLCs.

### 2.2 Study context

HLCs offer individual and group‐based lifestyle interventions focusing on the promotion of healthy dietary and physical activity habits. This interdisciplinary primary health‐care service provides educational self‐management interventions aimed at empowering people to manage their condition or health behaviour change. The HLC emphasizes the strengthening of physical, mental and social resources for health and self‐management based on a health promoting, preventive and salutogenic foundation. User involvement is a key principle that is enshrined in the legislation, and the interventions are based on a person‐centred approach, adjusted to service users’ needs, individual resources and self‐management skills.^28^ HPs, including physiotherapists, public health nurses, psychiatric nurses, nutritionists and bachelors in public health, provide the interventions. HLCs are easily accessible through direct contact or by referrals from general practitioners. The initial health conversation is based on each service user's needs and desire for help, after which a group‐based healthy diet course and/or physical activity sessions is offered. The healthy diet course consists of four to five two‐hour sessions with practical tasks and theory. Physical activity in the form of group‐based indoor and outdoor activities is offered two to three times a week. If desired, individual counselling is also available. An intervention lasts for three months with the possibility to extend it on two occasions.

### 2.3 Recruitments

The participants in this study were recruited from five different small and medium‐sized HLCs in Norway. The first author (ES) contacted HLC administrators and asked them to send an information letter about the study with an invitation to take part to both women and men who had participated in lifestyle courses. The inclusion criteria were persons aged 18 to 80 years who had contacted the HLC to obtain help to change their dietary and activity habits, afflicted by overweight or obesity and who were able to speak and understand the Norwegian language. Purposive sampling^34^ was used to identify participants for interview to ensure that the sample included individuals of various ages and both sexes from small (rural) and medium‐sized (urban) municipalities. The first author contacted all the service users who consented to participate. Before the individual interviews, precautions had been taken by reflecting on how to take care of the participants if the interview situation became unpleasant or challenging. The ethical guidelines in the Helsinki Declaration were followed. This study was registered and approved at the Norwegian Centre for Research Data (NSD) project number 48025.

### 2.4 Data collection

A thematic interview guide with follow‐up questions was developed by ES and ALH in line with Kvale and Brinckmann^35^ (Table 1). The form of the interviews was open, allowing for elaboration. Over a five‐month period in 2017, the first author (ES) conducted 13 individual in‐depth interviews with service users at five different HLCs in Western Norway. In accordance with the participants’ wishes, 11 interviews took place in their local HLC and two at a university campus. The purpose of the study was presented, and confidentiality was emphasized. The interviews, which lasted between 60 and 130 minutes, were audio‐taped and transcribed verbatim by ES. Information power as discussed by Malterud et al,^34^ e.g. a narrow aim, sample specificity, high‐quality dialogue and amount of information, guided the sample size.

**TABLE 1:** Thematic guide for individual interviews

Self‐management support:
Can you describe what you have experienced as beneficial support in the lifestyle interventions in HLCs?
What do you perceive as helpful for lifestyle change?
How was the information and support in the intervention adjusted to your needs?
What have given you strength to start or continue lifestyle change?
Can you describe your need for follow‐up in the future?
User involvement:
What do you understand by user involvement at the HLCs lifestyle interventions?
What is important for you regarding user involvement?
How did you get involved?
What give you a sense of being involved?
How were your opinions met?
Can you describe your own role in the involvement?

### 2.5 Analysis

Data were analysed by the analytical steps in Qualitative Content Analysis suggested by Graneheim & Lundman^36^ and Graneheim, Lindgren & Lundman.^37^ According to these authors, categories present the manifest, descriptive content of the text, while a theme presents the latent content, a thread of an underlying meaning on an interpretative level.^36^ ES was responsible for the analysis with input from all the co‐authors (GF, BSH, ALH). Table 2 shows the steps in the analytical process. To capture complete ideas and something important in relation to the research question, the main theme and themes have been labelled with a phrase or a sentence.^38^, ^39^

**TABLE 2:** Steps of analytical process

1. Open reading	The transcripts were read repeatedly to obtain an overall impression and discussed at group meeting between the authors
2. Identifying meaning units	The text was divided into meaning units by first author
3. Condensing meaning units	These meaning units were condensed into a more written style
4. Creating codes and categories	The condensed meaning units were further abstracted and labelled with a code by the first author (e.g., listening, understanding needs, modifying, tailoring, adjusting support, flexibility, openness)
5. Sorted codes and categories abstracted into sub‐themes	The codes were abstracted into tentative sub‐themes and continuously regrouped and discussed between the authors (e.g., the importance of flexibility and individualized support)
6. Formulating into latent theme	The sub‐themes were discussed, and the latent content was labelled with a sentence to capture context, complete ideas and reasoning
7. Interpretation into one main theme	In accordance with the hermeneutical methodological approach, the themes were interpreted into a main theme answering the aim of the study

## 3 RESULTS

A total of 13 service users, five men and eight women, were included in this study (Table 3). Some had been recommended lifestyle changes such as regular moderately intensive physical activity, healthy diet and weight reduction by their general practitioners. However, the majority had contacted the HLC on their own initiative because they wanted to lose weight and needed help to change their lifestyle habits. Several of the participants had contact with HLC and/or participated in physical activity group sessions for more than a year, and they still had this contact. The participants were either overweight (Body‐Mass Index [BMI] ≥ 25) or obese (BMI ≥ 30) and had additional diagnoses that put them at risk (Table 4).

**TABLE 3:** Participant characteristics

	Sex	Age	Occupational status	Proposal and/or referral from GP/ own initiative	Type of intervention	Duration of contact and participation period
1	Male	60‐69	Retired	Own	IHC, HDG	9 months
2	Female	60‐69	Disability pension	Own	IHC, HDG, PAG	2 years
3	Male	60‐69	Retired	Own and GP proposal	IHC, HDG, PAG	1‐2 years
4	Female	40‐49	Disability pension	Own, GP referral	IHC, HDG, PAG	2‐31/2 years
5	Male	30‐39	Unemployed	Own and GP proposal	IHC	9‐12 months
6	Female	50‐59	Disability pension	Own, GP referral	IHC, HDG, PAG	2 years
7	Female	40‐49	Employed 50%‐80%	Own	IHC, HDG, PAG	9‐12 months
8	Female	60‐69	Disability pension	Own, GP referral	IHC, HDG, PAG	2‐31/2 years
9	Male	60‐69	Unemployed/long‐term sick leave	Own and GP proposal	IHC, PAG	2‐31/2 years
10	Female	30‐39	Employed 50%‐80%	GP proposal and referral	IHC, HDG, PAG	1‐2 years
11	Female	30‐39	Unemployed	Own	IHC, HDG, PAG	3‐6 months
12	Female	40‐49	Employed 50%‐80%	GP proposal and referral	IHC, HDG, PAG	9‐12 months
13	Male	50‐59	Employed 50%‐80%	Own, GP proposal and referral	IHC, HDG, PAG	2‐31/2 years

Note

Abbreviations in Table 1: Individual Health Conversation (IHC), Healthy Diet in Groups (HDG), Physical Activity in Groups (PAG).

**TABLE 4:** Self‐reported challenges, strains and additional diagnoses (number of participants in brackets)

**One or several of somatic diagnosis**: Type 2 diabetes (3), cardiovascular disease (CVD) (4), chronic obstructive pulmonary disease (COPD) (2), coeliac disease (1), multi sclerosis (MS) (1), sleep apnoea (1), various chronic pain conditions (8), fibromyalgia (3), cancer (2)
**One or several of psychosocial strains and challenges**: Anxiety (3), depression (4), loss and grief (1), identity reactions (12), eating disorders (2), suicidal thoughts (2), alcohol abuse (1), isolation (6), financial difficulties (2)

The analysis resulted in one main theme and four themes compromising several sub‐themes related to the service users’ experience of beneficial self‐management support and user involvement when attending to lifestyle interventions in the HLC (Table 5).

**TABLE 5:** Main theme, themes and sub‐themes describing service users’ experiences of beneficial self‐management support and user involvement in the HLCs

*Main theme*: Regaining self‐esteem and dignity through active involvement and long‐term self‐worth support in partnership with others	
*Theme*	*Sub‐theme*
Self‐efficacy through active involvement and better perceived health	Being in control by having ownership of personal goals
	Responsibility by showing initiative and participating
	The significance of the effects of training
Valued through HPs acknowledgement, equality and individualized support	Knowledgeable health professionals increase trust and safety
	Feeling stronger by perceiving emotional support from interested and sensitive health professionals
	Sense of equality and worth through acknowledgement
	The importance of flexibility and individualized support
Increased motivation and self‐belief through peer support and fellowship	Encouragement and a sense of worth through peer support in an inclusive environment
	A sense of identity and fellowship through the sharing of experience
	Meaningfulness by obtaining structure and commitment
Maintenance of lifestyle change through accessibility and long‐term support	A need for continued awareness and focus
	A need for long‐term support to maintain lifestyle change
	The importance of accessibility

### 3.1 Regaining self‐esteem and dignity through active involvement and long‐term self‐worth support in partnership with others

Overall, the participants were taking personal responsibility for active involvement. They described help as supporting self‐worth and increasing their belief in self‐management. They perceived being strengthened and regaining self‐esteem and dignity by being invited to become involved in an equal partnership, built on a trustful relationship with competent HPs. User involvement was described as acknowledgement. Thus, acknowledgement can be the HPs’ ability to personalize and tailor self‐management support and lifestyle intervention to service users’ needs and everyday life. Participation in supervised groups and emotional support from peers increased motivation and self‐belief. Several of the participants expressed a need for long‐term support, and accessibility and long‐term support was critical to maintenance of lifestyle change.

#### 3.1.1 Self‐efficacy through active involvement and better perceived health

The participants described taking responsibility for the initiative to engage in the lifestyle interventions in the HLC. They described maintaining new habits, what they had managed to change in their diets, activity habits and how much weight they had lost. They showed willpower and discipline by describing how they performed even if they disliked it. Everyone had already experienced the positive effects of training such as improved strength, fitness and weight loss. Some experienced sleeping better, using less painkillers and that their knees and hips were better off during periods of active lifestyle and training. Feelings of progress and positive health outcomes made them enjoy activity and training. This experience of well‐being, more energy and strength gave them a sense of meaningfulness and motivation to continue lifestyle changes.

It's motivating to see the changes in my body fat, change in this abdominal fat and muscle mass, very motivating. I think quitting smoking and starting to work out is the best thing I`ve done… at least I feel much better with myself. (Male 60‐69).

Several of the participants experienced having developed a belief in their own ability to manage lifestyle change, be in control and also of being aware of and developing personal skills related to willpower, knowledge, understanding of consequences and using their own resources in the process of change (self‐management‐skills). Belief in their own ability (self‐efficacy) and experience of well‐being and support strengthened their motivation to continue implementing lifestyle changes.

#### 3.1.2 Valued through HPs acknowledgement, equality and individualized support

The participants experienced the first meeting at the HLC, the individual counselling and all the subsequent meetings with HPs as supportive, with an emphasis on confidentiality that led to a feeling of safety. Everyone noted that the HPs were highly competent and knowledgeable possessing both communication and relational skills. HPs created a positive and welcoming atmosphere, shared their knowledge, as well as inviting and encouraging service users to participate, which in turn led to a positive commitment. HPs were described as being helpful, sensitive, including everyone, having a positive personality and attitude, taking time to listen to the participants’ stories and showing genuine interest in them as individuals:

They exude a desire to be there for you, they are genuinely interested in you. They want you to participate… The openness and the way everyone are included have been the most important experience for me. The philosophy here is fantastic! (Male 60‐69).

The participants experienced being met, listened to and receiving emotional support in terms of a coherent and holistic approach. Feedback, encouragement and emotional support were very important for all the participants. They described this as ‘they build you up’, meaning giving them belief in own ability to manage, self‐respect and worth. This can be exemplified by one of the participants:

The way they do it…they encourage you to perform, everything is good enough, they build you up and you get a feeling of being able to manage. This is a great motivation for me, and I feel I am doing well even if I am not a world champion. (Female 40‐49).

The participants experienced that HPs gave them responsibility for choice, but motivated, encouraged and provided positive feedback on their choices in the process of change. HPs emphasized ‘raising awareness’ and the service users’ personal resources and skills, focusing on what they managed, not what they failed to accomplish. The service users’ views were respected, and their needs and values recognized. Being listened to, respected and acknowledged increased the feeling of being taken seriously and a sense of being in a partnership and equal relationship with the ‘experts’. This attitude shows respect for service users’ autonomy and is illustrated by one of the participants:

I experience that my opinions are at least as important as theirs (HPs), so I have a strong feeling of being met, seen and heard. (Female 60‐69).

The service users experienced being free to choose to participate in dietary interventions, activity interventions or both and to some degree the freedom to choose individual counselling, group interventions or both. HPs emphasized group participation, but there was an opportunity to just have individual consultations. The participants appreciated the opportunity to choose what is best for them:

What I appreciate is that they don't force you in one direction. They say ‘you're welcome. This is our menu. What do YOU want?’ (Male 60‐69).

HPs made adjustments due to service users’ personal needs and the participants highly appreciated the opportunity for follow‐up conversations when they experienced relapses. Everyone reported receiving individual practical guidance from HPs in the form of, for example, alternative exercises, how to avoid energy dense food and how to reward themselves. Flexibility and adjustment to service users’ needs stimulated engagement and involvement in the participants’ own change process and management, serving as an invitation to codetermination in issues related to the participants’ need for health care. This individualized adjustment was also important for the sense of worth, which was exemplified by one of the participants:

They listen to what you say. They don't overrule you! They just facilitate and adjust. They listen very carefully to your opinions and adjust according to your needs. Theytell you to listen to your body, try as much as you can and it's good enough. That made me feel safe. (Female 40‐49).

#### 3.1.3 Increased motivation and self‐belief through peer support and fellowship

When starting at the HLC, several of the participants experienced a sense of being unsafe and feeling uncomfortable in group sessions, and the thought of working out with others and exposing their big body and bad shape was frightening. After some time, most of the participants experienced the group as a safe place. They experienced inclusion, an atmosphere of humour, respect and acknowledgement, as well as a safe place to express personal views. Getting inspiration from, sharing experiences and being supported and encouraged by the other participants were essential for endurance and motivation:

I prefer group training because it's so difficult to do the exercises alone… the motivation in the spinning sessions when your fellow participants tell you how well you are doing…the motivation and the encouragement that's what group training is all about and it keeps you going. (Female 30‐39).

In the group sessions, the social and emotional support, fellowship, the feeling of togetherness and creation of new friendships became more and more important for participation, joy, well‐being and meaningfulness in everyday life and served as a motivation for the continuation of change:

Participating in the training groups is like balsam to me. I can't do without it. It's not only the exercise but the whole group. The whole group contributes…. We care about each other and you get a feeling of being wanted and your presence is appreciated. It makes me feel so good. (Female 30‐39).

The participants also described both a need for structure and a sense of commitment to meet and participate in relation to the other participants and to themselves. Several of the participants were unemployed, retired or on a disability pension, thus participating in diet groups and especially physical activity groups helped to create structure, routines and meaning in everyday life:

The group is a commitment. So simple and so important! When you don't have a job to go to, it's important to get up in the morning, have something to do, have someone to meet, to be together in a group, training… it means everything for my mental health…to have this fellowship, meeting people with a positive attitude. (Female 40‐49).

#### 3.1.4 Maintenance of lifestyle change through accessibility and long‐term support

The service users described a need for someone to contact when they experience adversity and relapse. They considered that the HCL was a locally situated and easily accessible (low threshold) health‐care service that was easy to contact if they needed something, which created a sense of safety. The participation period for these service users, ranged from 3 to 42 months (Table 3), depending on individual needs and availability. One of the participants expressed:

It's very important to have someone to contact when you experience adversity. I have sent a request to my provider at the HLC and she always found time for me to have a talk about what is happening and how to manage it. The follow‐up is the best experience, from the first health conversation and now, two years later, getting support and encouragement when I need it. (Male 50‐59).

Some of the participants described needing someone to ‘put you on track again’ when relapses and old habits occur. The service users expressed a need for continuing participation in groups and follow‐up by HPs. Some expressed a desire for permanent follow‐up. Everyone experienced that HLCs offered a low‐cost intervention, stretching over a long period of time, which is important for letting the change process take place:

It is so important that the interventions and the activity group stretches over a period of time and that this is a health‐care service that lasts and gives me the opportunity to apply for a new period. (Female 60‐69).

## 4 DISCUSSION

HLCs offer self‐management interventions for persons in need of support for lifestyle change, with the intention of empowering the participants to achieve self‐management and improved health. The aim of this study was to explore beneficial self‐management support and user involvement for persons afflicted by overweight or obesity attending lifestyle interventions in HLCs. In the following, we will discuss the results in relation to previous research within this field and in the context of HLC and primary care. The main theme and themes will guide the discussion.

### 4.1 Regaining self‐esteem and dignity through active involvement and long‐term self‐worth support in partnership with others

Service user's active involvement and long‐term self‐worth support in partnership with HPs and fellow participants seem to be an overall means to achieve individual empowerment, user involvement and self‐management. We suggest that acknowledgement and individualized support from competent HPs promote self‐worth and participation, which are a prerequisite for user involvement and again for self‐management. These findings support the connection between self‐management support and user involvement and that these may be considered synonyms and overlapping in chronic diseases.^13^ Over time, acknowledgement, equality and self‐worth support may contribute to a feeling of dignity. Previous studies on HLC participants’ perceptions of seeking help for lifestyle change show that persons afflicted by overweight or obesity experience emotional distress and search for change and dignity.^33^, ^40^ How one looks at her/himself is an existential question of identity. Self‐worth support may lead to an increased feeling of being a valuable person, and belief in oneself.

The service users experience *self‐efficacy through active involvement and better perceived health*. This indicates that individual elements influencing user involvement and self‐management were related to the service users’ motivation. Perceived control and self‐efficacy are important elements in individual empowerment,^41^, ^42^ and an important goal of the interventions in HLCs.^28^ We suggest that taking the initiative to change and to participate in HLC shows autonomous motivation. Previous research confirms that a participant whose motivation was more autonomous would attend intervention programmes more regularly.^43^ Autonomous motivation, self‐efficacy, as well as self‐regulation skills and a positive body image are suggested to be the best individual psychological mechanisms for successful weight management and physical activity outcomes.^44^, ^45^ Self‐efficacy focused education would probably enhance self‐efficacy, regulate self‐management behaviours, increase knowledge and improve health‐related quality of life.^46^ Belief in one's own ability and self‐esteem are important concepts in Antonovsky's salutogenic approach and theory of general resistance resources.^47^ Based on the salutogenic approach in HLC, HPs should continue to provide emotional self‐worth support in order to promote self‐efficacy as a means for self‐management, thus achieving the key goal of educational interventions.^4^, ^6^ This includes personal responsibility for active involvement and a desire to lose weight and improve physical strength, health and well‐being. Experiencing positive effects of training and receiving beneficial support seemed to influence the participants motivation and initiation of activity. Better perceived health, feelings of progress, strength and well‐being made most of our participants enjoy physical activity and gave them a sense of meaningfulness, belief in their own ability and motivation to continue with lifestyle change. Previous experience and control are key factors for self‐efficacy, as control is related to the expectations of management.^48^

The present study reveals that the service users are *valued through HPs acknowledgement, equality and individual support*. The participants experienced a strong partnership with their providers and described that emotional support, acknowledgement and having a sense of equality in a partnership with HPs create a sense of worth and are essential for the service user's involvement in their process of change. Previous studies describe that a trustworthy relationship between service users and HPs seems to be based on respect, trust and acknowledgement.^32^, ^49^, ^50^ Acknowledgement and a sense of equality are described by our participants as a feeling of being listened to and taken seriously. This is consistent with other studies of lifestyle interventions, showing that acknowledgement, equality and individualization are core elements of user involvement.^32^, ^51^, ^52^ Thus, the importance of trained HPs who possess effective communication skills, and are competent, confident and supportive was highlighted.^32^, ^49^, ^53^ In our study, HPs’ flexibility and ability to personalize and tailor self‐management support and lifestyle intervention to the service users’ needs and everyday life was important for their participation and adherence to the intervention programme. Other studies from patient education programmes and lifestyle consultations in primary care confirm that HPs’ ability to be observant and individualize and tailor interventions to the service users’ values, needs and situation is essential, which underscores the necessity of a person‐centred approach.^32^, ^49^, ^53^, ^54^ Client‐centred therapy emphasizes acknowledgement and a trustful relationship as fundamental in therapy and behavioural change processes.^55^

*Increased motivation and self‐belief through peer support and fellowship* is demonstrated by the service users’ experience of the value of being included, group affiliation and a common identity. Group‐based interventions are characterized by opportunities for social interactions and support from others who are experiencing similar challenges. The group dynamic during patient education interventions might be more important for improving self‐management skills than the actual content of the programme.^56^ The social and emotional support in the fellowship with peers, the feeling of togetherness and the creation of new relationships and friendships became important for well‐being and meaningfulness in everyday life and as well as serving as a motivation for the continuation of change. This is also supported by studies from Norwegian primary care, where service users argued that the group‐based approach would improve social peer support, which could have a positive impact on participants’ well‐being and subsequently on self‐management.^57^ Learning from other patients’ experiences, being with peers, having a feeling of belonging and being with equals or people who understand you are described as the key to success in diabetes patient education.^58^ Our study supports these findings and we believe that the sense of belonging and identity may be especially important for our participants, taking into account the stigmatization experienced by persons with overweight or obesity.^27^, ^40^, ^59^ A Norwegian study shows that group‐based diabetes self‐management education improved self‐management skills and psychosocial outcomes such as self‐efficacy, and the authors suggest that other factors such as peer identification, normalization and group interactions are the ‘active ingredients’ and as such substantially influence the effectiveness of group‐based education interventions for the management of type 2 diabetes.^60^ Future research should explore these factors in weight self‐management for persons afflicted by overweight or obesity.

*Maintenance of lifestyle change through accessibility and long‐term support* is evident through the service users concerns about how they would manage on their own after the conclusion of the HLC intervention. Most of them expressed a desire for continued or prolonged follow‐up by competent HPs in the HLC, highlighting the value of an easily accessible, locally based health‐care service. A major challenge in the treatment of overweight and obesity is the long‐term maintenance of weight loss.^20^, ^61^ The reasons for high relapse rates in overweight and obesity treatment are complex and not fully understood. Nevertheless, there is a considerable amount of literature documenting that people need further follow‐up after participation in a lifestyle interventions ^21^, ^62^ and comprehensive evidence of the necessity for long‐term support or prolonged contact between HPs and service users to enable weight loss maintenance.^17^, ^22^, ^61^, ^63^ Frequent long‐term treatment and user‐HP contact, for example provided in group sessions, are perhaps the most successful methods for preventing weight regain.^20^, ^63^, ^64^ Over the last 20‐30 years, there has been an extensive amount of research that investigated long‐term weight loss, including professional contact and relapse prevention. These studies demonstrate that successful long‐term management of obesity may require maintenance programmes involving years rather than months of follow‐up care and extended treatment in the form of weekly or bi‐weekly individual or group therapy sessions^17^, ^22^ in person, by phone or internet.^65^ On the other hand, prolonged follow‐up involves a cost. It is reasonable to believe that HLC participants need further support and motivation to continue regularly monitoring their food intake and physical activity in order to maintain their lifestyle change and self‐management. Our findings demonstrate that several of the participants continued to take part, especially in activity group sessions, after the conclusion of their three assigned periods (Table 3). We believe that some HPs recognize the need for long‐term follow‐up and in some way are ‘gaming the system’ by letting the participants continue to attend in group sessions. By so doing, they recognize each service user's need for continued support, which can be seen as adjusted and individualized health‐care support, as well as a shared responsibility of partnership. Given the chronic nature of obesity, extended care may be necessary to achieve long‐term health benefits,^52^, ^61^ but first and foremost, obesity should be recognized as a chronic condition that requires lifelong support.^21^, ^22^ The considerable amount of literature on self‐management in chronic conditions like type 2 diabetes and cardiovascular diseases, in contrast to the sparse literature on overweight and obesity may be explained as a lack of recognition of overweight and obesity as a chronic disease. The World Obesity Federation considers obesity to be a chronic relapsing disease.^2^, ^66^ However, recognition of obesity as a disease is by no means universal.^67^ HPs play a critical role in facilitating long‐term changes and follow‐up after lifestyle interventions.^65^ We suggest that cost‐effective follow‐up programmes, maybe over years, should be developed, including long‐term self‐care strategies with a supportive design and practice to promote self‐esteem and dignity. Further studies should also focus on methods to improve these programmes regarding social support, for example recruitment of participants with friends or family to safeguard the necessary long‐term support. There is a need to investigate HPs’ role and understanding of these matters to fully understand how self‐management support and user involvement are beneficial for self‐management and lifestyle change in HLCs.

## 5 METHODOLOGICAL CONSIDERATIONS

The trustworthiness of the findings is related to confidence in the analysis^68^ and to the researchers’ preunderstanding and interpretation of the statements made by the service users.^69^ We argue that the trustworthiness of our findings was strengthened by systematically analysing the data using inductive coding and categorization.^70^ The first author (ES) performed the analysis and interpretation of the data, while the co‐authors (GF, BSH, ALH) critically reviewed the interpretations. Discussion of the sub‐themes and themes on several occasions over a period of time was a process aimed at finding the most appropriate interpretation and increasing the credibility of the findings. ES conducted the interviews and in order to minimize potential bias, the co‐authors read all the transcribed interviews. The researchers’ various disciplinary backgrounds and clinical experience as a psychiatric nurse (ALH), a public health nurse (ES, BSH), patient education (GF) and intensive care (BSH) enriched the analysis and interpretation, thereby increasing the trustworthiness. The reporting quality in the present paper was cross‐checked to comply with the consolidated criteria for reporting qualitative studies using the 32‐item COREQ checklist.^71^

The strengths of the present study include the richness of each semi‐structured in‐depth interview. The variation in age, gender, background and current situation, in addition to the collection of data from five different HLCs in both rural and urban municipalities, reflects multiple realities and practices, which might increase the transferability to other settings.

However, some methodological limitations should be addressed. HPs’ recruitment of participants could be influenced by their knowledge of service users who were especially satisfied. The self‐selection of volunteers to participate and the service users’ opportunity to participate in HLC interventions in the daytime (due to their employment situation) may have influenced their descriptions of user involvement and satisfaction (structure and social support). We have no data on those who declined to take part in the study, or those who were prevented from participating for various reasons. However, as the aim of the study concerned experience of beneficial self‐management support and user involvement (and not useless support and barriers to participation), the participants recruited were therefore suitable.

## 6 CONCLUSION

Our findings suggest that service user's active involvement and long‐term self‐worth support in partnership with others seem to promote self‐management and user involvement. Acknowledgement from HPs in HLCs, self‐management support tailored to service users’ needs, and peer support in supervised group sessions seems to be important mechanisms for increasing user involvement, self‐efficacy and self‐esteem, leading to dignity and individual empowerment. We believe that lifestyle change is not simply a question of individual autonomous motivation and willpower, but primarily concerns relational, emotional and social support. Long‐term self‐worth support from significant others seems to be some of the successful ingredients to lifestyle change. A collectivist perspective can integrate the importance of significant others, involvement and shared responsibility. Motivating participants to participate in HLC interventions together with a friend or a partner may lead to more independence, ‘self’‐management and lasting lifestyle changes. Recognizing overweight and obesity as a chronic condition in line with diabetes type 2 etc and providing long‐term support, maybe over many years for those in need, will strengthen the ability of HLCs to provide beneficial ‘self’‐management support to persons afflicted by overweight or obesity.

## CONFLICTS OF INTEREST

The authors declare that they have no competing interests.

## Data Availability

The data set used and analysed during the current study is available from the corresponding author on reasonable request.

## References

[1] World Health Organization: Noncommunicable diseases Fact Sheet. https://www.who.int/en/news‐room/fact‐sheets/detail/noncommunicable‐diseases 2018. Accessed 18.01. 2019.

[2] World Health Organization: Obesity and overweight Fact Sheet. https://www.who.int/en/news‐room/fact‐sheets/detail/obesity‐and‐overweight 2018. Accessed 23.01.2019.

[3] Stenberg U , Vågan A , Flink M , et al. Health economic evaluations of patient education interventions a scoping review of the literature. Patient Educ Couns. 2018;101(6):1006‐1035. 10.1016/j.pec.2018.01.006 29402571

[4] Bodenheimer T , Lorig K , Holman H , Grumbach K . Patient self‐management of chronic disease in primary care. JAMA. 2002;288(19):2469‐2475. 10.1001/jama.288.19.2469 12435261

[5] Barlow J , Wright C , Sheasby J , Turner A , Hainsworth J . Self‐management approaches for people with chronic conditions: a review. Patient Educ Couns. 2002;48(2):177‐187. 10.1016/s0738-3991(02)00032-0 12401421

[6] Lorig KR , Holman HR . Self‐management education: history, definition, outcomes, and mechanisms. Ann Behav Med. 2003;26(1):1‐7. 10.1207/S15324796ABM2601_01 12867348

[7] Thille P , Ward N , Russell G . Self‐management support in primary care: Enactments, disruptions, and conversational consequences. Soc Sci Med. 2014;108:97‐105. 10.1016/j.socscimed.2014.02.041 24632054

[8] Vrangbaek K . Patient involvement in Danish health care. J Health Organ Manag. 2015;29(5):611‐624. 10.1108/jhom-01-2015-0002 26222880

[9] Dent M , Pahor M . Patient involvement in Europe–a comparative framework. J Health Organ Manag. 2015;29(5):546‐555. 10.1108/jhom-05-2015-0078 26222875

[10] The Norwegian Ministry of Health and Care Service: Report to the Storting Meld. St. 26 (2014‐2015), The primary health and care services of tomorrow – localised and integrated, (White Paper). https://www.regjeringen.no/en/dokumenter/meld.‐st.‐26‐20142015/id2409890/?q=meld 2015. Accessed 12.01.2019.

[11] World Health Organization: Health Promotion Glossary. https://www.who.int/healthpromotion/about/HPR%20Glossary%201998.pdf?ua=1 1998. Accessed 21.10.18.

[12] Omeni E , Barnes M , MacDonald D , Crawford M , Rose D . Service user involvement: impact and participation: a survey of service user and staff perspectives. BMC Health Serv Res. 2014;14(1):491 10.1186/s12913-014-0491-7 25344210 PMC4212124

[13] Greenhalgh T . Patient and public involvement in chronic illness: beyond the expert patient. BMJ. 2009;338:b49 10.1136/bmj.b49 19223339

[14] Artinian NT , Fletcher GF , Mozaffarian D , et al. Interventions to promote physical activity and dietary lifestyle changes for cardiovascular risk factor reduction in adults. A scientific statement from the American Heart Association. Circulation. 2010 10.1161/CIR.0b013e3181e8edf1 PMC6893884 20625115

[15] Greaves CJ , Sheppard KE , Abraham C , et al. Systematic review of reviews of intervention components associated with increased effectiveness in dietary and physical activity interventions. BMC Public Health. 2011;11(1):119 10.1186/1471-2458-11-119 21333011 PMC3048531

[16] Dombrowski SU , Knittle K , Avenell A , Araujo‐Soares V , Sniehotta FF . Long term maintenance of weight loss with non‐surgical interventions in obese adults: systematic review and meta‐analyses of randomised controlled trials. BMJ. 2014;348:g2646 10.1136/bmj.g2646 25134100 PMC4020585

[17] Perri MG , Ariel‐Donges AH . Maintenance of weight lost in behavioral treatment of obesity In: Wadden TA, Bray GA, eds. Handbook of Obesity Treatment. New York: The Gilford Press; 2018: 393.

[18] Lagger G , Pataky Z , Golay A . Efficacy of therapeutic patient education in chronic diseases and obesity. Patient Educ Couns. 2010;79(3):283‐286. 10.1016/j.pec.2010.03.015 20413242

[19] Albano MG , Golay A , De Andrade V , Crozet C , d’Ivernois J‐F . Therapeutic patient education in obesity: analysis of the 2005–2010 literature. Educ Ther Patient/Ther Patient Educ. 2012;4(2):S101‐S110. 10.1051/tpe/2012011

[20] Montesi L , El Ghoch M , Brodosi L , Calugi S , Marchesini G , Dalle Grave R . Long‐term weight loss maintenance for obesity: a multidisciplinary approach. Diabetes Metab Syndr Obes. 2016;9:37–46. 10.2147/DMSO.S89836 27013897 PMC4777230

[21] Martins C . Wight loss maintenance ‐ a tortuos path. Indremedisineren, https://indremedisinerenno/2018/02/weight‐loss‐maintenance‐a‐tortuous‐path/ 2018, (04:2017).

[22] Perri MG . The maintenance of treatment effects in the long‐term management of obesity. Clin Psychol. 1998;5(4):526‐543. 10.1111/j.1468-2850.1998.tb00172.x

[23] Booth HP , Prevost TA , Wright AJ , Gulliford MC . Effectiveness of behavioural weight loss interventions delivered in a primary care setting: a systematic review and meta‐analysis. Fam Pract. 2014;31(6):643‐653. 10.1093/fampra/cmu064 25298510 PMC4240316

[24] Kelly MP , Barker M . Why is changing health‐related behaviour so difficult? Public Health. 2016;136:109‐116. 10.1016/j.puhe.2016.03.030 27184821 PMC4931896

[25] Phelan SM , Burgess DJ , Yeazel MW , Hellerstedt WL , Griffin JM , van Ryn M . Impact of weight bias and stigma on quality of care and outcomes for patients with obesity. Obes Rev. 2015;16(4):319‐326. 10.1111/obr.12266 25752756 PMC4381543

[26] Spooner C , Jayasinghe UW , Faruqi N , Stocks N , Harris MF . Predictors of weight stigma experienced by middle‐older aged, general‐practice patients with obesity in disadvantaged areas of Australia: a cross‐sectional study. BMC Public Health. 2018;18(1):640 10.1186/s12889-018-5556-9 29783962 PMC5963137

[27] Puhl RM , Quinn DM , Weisz BM , Suh YJ . The role of stigma in weight loss maintenance among US adults. Ann Behav Med. 2017;51(5):754‐763. 10.1007/s12160-017-9898-9 28251579

[28] The Norwegian Directorate of Health: Guidelines for Municipal Healthy Life Centers. https://helsedirektoratet.no/Lists/Publikasjoner/Attachments/53/IS‐1896‐Frisklivsveileder.pdf 2016. Accessed 24.11. 2018.

[29] Samdal GB , Meland E , Eide GE , et al. The Norwegian Healthy Life Centre Study: a pragmatic RCT of physical activity in primary care. Scand J Public Health. 2019;47(1):18‐27. 10.1177/1403494818785260 30074437

[30] Lerdal A , Celius EH , Pedersen G . Prescribed exercise: a prospective study of health‐related quality of life and physical fitness among participants in an officially sponsored municipal physical training program. J Phys Act Health. 2013;10(7):1016‐1023. 10.1123/jpah.10.7.1016 23136380

[31] Følling IS , Kulseng B , Midthjell K , Rangul V , Helvik AS . Individuals at high risk for type 2 diabetes invited to a lifestyle program: characteristics of participants versus non‐participants (the HUNT Study) and 24‐month follow‐up of participants (the VEND‐RISK Study). BMJ Open Diabetes Res Care. 2017;5(1):e000368 10.1136/bmjdrc-2016-000368 PMC5574427 28878932

[32] Sagsveen E , Rise MB , Grønning K , Bratås O . Individual user involvement at Healthy Life Centres: a qualitative study exploring the perspective of health professionals. Int J Qual Stud Health Well‐being. 2018;13(1):1492291 10.1080/17482631.2018.1492291 30010499 PMC6052421

[33] Følling IS , Solbjør M , Helvik A‐S . Previous experiences and emotional baggage as barriers to lifestyle change ‐ a qualitative study of Norwegian Healthy Life Centre participants. BMC Fam Pract. 2015;16(1):73 10.1186/s12875-015-0292-z 26100276 PMC4476174

[34] Malterud K , Siersma VD , Guassora AD . Sample size in qualitative interview studies: guided by information power. Qual Health Res. 2016;26(13):1753‐1760. 10.1177/1049732315617444 26613970

[35] Kvale S , Brinkmann S . Interviews. Learning the Craft of Qualitative Research Interviewing. 3rd ed California, USA: SAGE Publications Inc.; 2015.

[36] Graneheim UH , Lundman B . Qualitative content analysis in nursing research: concepts, procedures and measures to achieve trustworthiness. Nurse Educ Today. 2004;24(2):105‐112. 10.1016/j.nedt.2003.10.001 14769454

[37] Graneheim UH , Lindgren B‐M , Lundman B . Methodological challenges in qualitative content analysis: a discussion paper. Nurse Educ Today. 2017;56:29‐34. 10.1016/j.nedt.2017.06.002 28651100

[38] Sandelowski M , Leeman J . Writing usable qualitative health research findings. Qual Health Res. 2012;22(10):1404‐1413. 10.1177/1049732312450368 22745362

[39] Braun V , Clarke V . Using thematic analysis in psychology. Qual Res Psychol. 2006;3(2):77‐101. 10.1191/1478088706qp063oa

[40] Salemonsen E , Hansen BS , Førland G , Holm AL . Healthy Life Centre participants’ perceptions of living with overweight or obesity and seeking help for a perceived “wrong” lifestyle ‐ a qualitative interview study. BMC Obes. 2018;5(1):42 10.1186/s40608-018-0218-0 30546910 PMC6282310

[41] World Health Organization: First International Conference on Health Promotion, Ottawa, 21. November 1986. http://www.who.int/healthpromotion/conferences/previous/ottawa/en/ 1986. Accessed 12.06.17.

[42] Gutiérrez LM . Working with women of color:an empowerment perspective. Soc Work. 1990;35(2):149 10.1093/sw/35.2.149

[43] Williams GC , Grow VM , Freedman ZR , Ryan RM , Deci EL . Motivational predictors of weight loss and weight‐loss maintenance. J Pers Soc Psychol. 1996;70(1):115 10.1037//0022-3514.70.1.115 8558405

[44] Teixeira PJ , Carraça EV , Marques MM , et al. Successful behavior change in obesity interventions in adults: a systematic review of self‐regulation mediators. BMC Med. 2015;13(1):84 10.1186/s12916-015-0323-6 25907778 PMC4408562

[45] Teixeira PJ , Silva MN , Mata J , Palmeira AL , Markland D . Motivation, self‐determination, and long‐term weight control. Int J Behav Nutr Phys Act. 2012;9(1):22 10.1186/1479-5868-9-22 22385818 PMC3312817

[46] Jiang X , Wang J , Lu Y , Jiang H , Li M . Self‐efficacy‐focused education in persons with diabetes: a systematic review and meta‐analysis. Psychol Res Behav Manag. 2019;12:67 10.2147/prbm.s192571 30774486 PMC6357887

[47] Antonovsky A . Unraveling the Mystery of Health. How People Manage Stress and Stay Well. San Francisco: Jossey‐Bass; 1987.

[48] Bandura A . Self‐efficacy: The Exercise of Control. New York: Freeman; 1997.

[49] Svavarsdóttir MH , Sigurdardottir AK , Steinsbekk A . What is a good educator? A qualitative study on the perspective of individuals with coronary heart disease. Eur J Cardiovasc Nurs. 2016;15(7):513‐521. 10.1177/1474515115618569 26588939

[50] Sagsveen E , Rise MB , Grønning K , Westerlund H , Bratås O . Respect, trust and continuity: a qualitative study exploring service users’ experience of involvement at a Healthy Life Centre in Norway. Health Expect. 2019;22(2):226‐234. 10.1111/hex.12846 30472770 PMC6433315

[51] Rise MB , Solbjør M , Lara MC , Westerlund H , Grimstad H , Steinsbekk A . Same description, different values. How service users and providers define patient and public involvement in health care. Health Expect. 2011;16(3):266‐276. 10.1111/j.1369-7625.2011.00713.x 21838833 PMC5060665

[52] Salemonsen E , Førland G , Hansen BS , Holm AL . Understanding beneficial self‐management support and the meaning of user involvement in lifestyle interventions: a qualitative study from the perspective of healthcare professionals. BMC Health Serv Res. 2020;20(88):1‐12. 10.1186/s12913-020-4951-y PMC7003436 32024505

[53] Klein J , Brauer P , Royall D , et al. Patient experiences of a lifestyle program for metabolic syndrome offered in family medicine clinics: a mixed methods study. BMC Fam Pract. 2018;19(1):148 10.1186/s12875-018-0837-z 30170544 PMC6119314

[54] Walseth LT , Abildsnes E , Schei E . Patients' experiences with lifestyle counselling in general practice: a qualitative study. Scand J Prim Health Care. 2011;29(2):99‐103. 10.3109/02813432.2011.553995 21294605 PMC3347940

[55] Rogers CR . The foundations of the person‐centered approach. Dialectics and Humanism. 1981;8(1):5‐16. 10.5840/dialecticshumanism19818123

[56] Nossum R , Rise MB , Steinsbekk A . Patient education–Which parts of the content predict impact on coping skills? Scand J Public Health. 2013;41(4):429‐435. 10.1177/1403494813480279 23474954

[57] Solberg HS , Steinsbekk A , Solbjør M , Granbo R , Garåsen H . Characteristics of a self‐management support programme applicable in primary health care: a qualitative study of users’ and health professionals’ perceptions. BMC Health Serv Res. 2014;14(1):562 10.1186/s12913-014-0562-9 25380808 PMC4229612

[58] Cooper H , Booth K , Gill G . Patients’ perspectives on diabetes health care education. Health Educ Res. 2003;18(2):191‐206. 10.1093/her/18.2.191 12729178

[59] Puhl RM , Heuer CA . The stigma of obesity: a review and update. Obesity. 2009;17(5):941‐964. 10.1038/oby.2008.636 19165161

[60] Steinsbekk A , Rygg L , Lisulo M , Rise MB , Fretheim A . Group based diabetes self‐management education compared to routine treatment for people with type 2 diabetes mellitus. A systematic review with meta‐analysis. BMC Health Serv Res. 2012;12(1):213 10.1186/1472-6963-12-213 22824531 PMC3418213

[61] Ross Middleton K , Patidar S , Perri M . The impact of extended care on the long‐term maintenance of weight loss: a systematic review and meta‐analysis. Obes Rev. 2012;13(6):509‐517. 10.1111/j.1467-789x.2011.00972.x 22212682

[62] Evans EH , Sainsbury K , Kwasnicka D , Bolster A , Araujo‐Soares V , Sniehotta FF . Support needs of patients with obesity in primary care: a practice‐list survey. BMC Fam Pract. 2018;19(1):6 10.1186/s12875-017-0703-4 29310572 PMC5759249

[63] Jiandani D , Wharton S , Rotondi MA , Ardern CI , Kuk JL . Predictors of early attrition and successful weight loss in patients attending an obesity management program. BMC Obes. 2016;3(1):14 10.1186/s40608-016-0098-0 26966544 PMC4784380

[64] Butryn ML , Webb V , Wadden TA . Behavioral treatment of obesity. Psychiatr Clin North Am. 2011;34(4):841‐859. 10.1016/j.psc.2011.08.006 22098808 PMC3233993

[65] Middleton KR , Anton SD , Perri MG . Long‐term adherence to health behavior change. Am J Lifestyle Med. 2013;7(6):395‐404. 10.1177/1559827613488867 27547170 PMC4988401

[66] Bray G , Kim K , Wilding J . Obesity: a chronic relapsing progressive disease process. A position statement of the World Obesity Federation. Obes Rev. 2017;18(7):715‐723. 10.1111/obr.12551 28489290

[67] Sharma AM , Campbell‐Scherer DL . Redefining obesity: beyond the numbers. Obesity. 2017;25(4):660‐661. 10.1002/oby.21801 28349662

[68] Polit DF , Beck CT . Nursing Reseach ‐ Generating and Assessing Evidence for Nursing Practice. 10th ed Philadelphia, USA: Wolters Kluwer Health; 2017.

[69] Fleming V , Gaidys U , Robb Y . Hermeneutic research in nursing: developing a Gadamerian‐based research method. Nurs Inq. 2003;10(2):113‐120. 10.1046/j.1440-1800.2003.00163.x 12755860

[70] Hsieh H‐F , Shannon SE . Three approaches to qualitative content analysis. Qual Health Res. 2005;15(9):1277‐1288. 10.1177/1049732305276687 16204405

[71] Tong A , Sainsbury P , Craig J . Consolidated criteria for reporting qualitative research (COREQ): a 32‐item checklist for interviews and focus groups. Int J Qual Health Care. 2007;19(6):349‐357. 10.1093/intqhc/mzm042 17872937

